# Implementation of Artificial Intelligence in Colonoscopy Practice in Japan

**DOI:** 10.31662/jmaj.2024-0133

**Published:** 2024-08-30

**Authors:** Masashi Misawa, Shin-ei Kudo, Yuichi Mori

**Affiliations:** 1 Digestive Disease Center, Showa University Northern Yokohama Hospital, Yokohama, Japan; 2 Clinical Effectiveness Research Group, University of Oslo and Oslo University Hospital, Oslo, Norway; 3 Gastroenterology Section, Department of Transplantation Medicine, Oslo University Hospital, Oslo, Norway

**Keywords:** colonoscopy, artificial intelligence, computer-aided diagnosis

## Abstract

This review outlines the implementation of artificial intelligence (AI) into colonoscopy procedures which includes its history, processes, and challenges. We highlight the importance of the collaborative effort between medical and computer science researchers in the development of AI tools in colonoscopy, particularly focusing on the roles of computer-aided detection (CADe) and computer-aided characterization (CADx) in a real time analysis of colonoscopy videos. Some of the proposed technologies are considered to improve the important clinical outcomes of patients such as adenoma detection rate in colonoscopy. Regulatory approval is considered mandatory before introducing AI tools into the market owing to the potential risks associated with the introduction of AI tools in healthcare. We share the experience of obtaining regulatory approval for EndoBRAIN in Japan, emphasizing the challenges in establishing examination criteria and performance levels at the period. Reimbursement is also identified as necessary for the widespread adoption of medical innovation. With the introduction of reimbursement for a CADe tool in Japan in 2024, we expect to accelerate implementation of AI in colonoscopy in general. Despite regulatory approval and reimbursement, concerns are raised with regard to the assessment of the balance between benefits and harms of AI in colonoscopy. Questions about its impact on cancer prevention, healthcare burden, patient acceptance, and effectiveness across different populations remain unsolved. The lack of clinical guidelines for AI in colonoscopy emphasizes the need for a rigorous assessment of available evidence in optimizing the adoption of AI in colonoscopy practice. While it is always exciting to strive for medical innovation, ensuring rigorous evaluation to optimize patient care is mandatory to improve the quality of health and society.

## 1. Introduction

Artificial intelligence (AI) has emerged as a promising tool in colonoscopy, revolutionizing the field with its potential in improving detection, diagnosis, and treatment of colorectal diseases. The history of AI in colonoscopy can be traced back to the early 2000s when researchers in the computer engineering field began the exploration of its application in image analysis and pattern recognition. Since then, significant advancements in machine learning, computer vision, and deep learning algorithms have paved the way for the development of AI-driven technologies tailored to colonoscopy, including computer-aided detection and characterization of colorectal polyps and lesions which works in real time during colonoscopy. We need to acknowledge the fact that remarkable and intensive collaboration between medical researchers (physician researchers) and computer scientists has played a significant role in identifying possible challenges in the implementation of this advanced technology, which has eventually contributed to early introduction of AI tools in colonoscopy practice. In the present paper, we review how the AI in colonoscopy has gone through challenges associated with its introduction to healthcare venue, including hurdles particularly in regulatory approvals and acquisition of reimbursement rates.

## 2. What Is Computer-Aided Detection and Diagnosis in Colonoscopy?

As we mentioned, computer-aided detection (CADe) and computer-aided characterization (CADx) have been the two main pillars in AI in colonoscopy in the last couple of decades.

CADe focuses on the detection of abnormalities in colonoscopy videos. AI algorithms analyze the visual data in real time, flagging suspicious regions which may indicate the presence of polyps or lesions. These flagged regions are then reviewed by an endoscopist for further evaluation, leading to comprehensive decision-making process between human and AI. More than 20 randomized trials showed that the use of CADe contributes to a significant increase in adenoma detection rate, a proportion of colonoscopies in which one or more adenoma (neoplasm) is detected ^(1)^．This increment has decent clinical implication because an increased rate of adenoma detection is closely associated with improved cancer prevention effect ^(2)^．A recent microsimulation study suggested a possibility of an absolute 5% reduction in colorectal cancer incidence with CADe being fully implemented in the US colonoscopy screening program ^(3)^.

On the other hand, CADx involves the characterization or classification of detected abnormalities (mainly polyps) into histological categories like adenomatous or hyperplastic polyps, as well as differentiating between benign and malignant lesions. The accuracy of CADx systems varies depending on the complexity of the classification task and the quality of the training data. Some studies have reported accuracy for CADx ranging from 70% to 90%, demonstrating the algorithms’ ability in assisting lesion characterization ^(4), (5)^．Unlike the CADe field, there have been no prospective studies, which show added value of CADx in terms of accuracies, raising the need for further investigation in this area ^(6), (7), (8)^.

## 3. Regulatory Approval

Regulatory approval is mandatory for these AI tools to be used in healthcare of most countries in the world including Japan. It is because the use of an AI in medicine is generally considered to accompany possible risks, which may harm patients. For example, there is a risk of overlooking polyps when using CADe. Missed adenomas can be a cause of colorectal cancer in the long run. Similarly, CADx may become a trigger of overlooking adenomas since CADx’s incorrect interpretation of adenomas can lead to nonremoval of such neoplasia. Therefore, it is considered important to test the balance between benefits and harms before these medical devices are introduced into the market. Usually, regulatory bodies (e.g., Pharmaceuticals Medical Device Agency [PMDA] in Japan) are responsible in overseeing this testing process.

Here, we would like to share our experience in overcoming regulatory clearance processes in Japan. Considering that our team was the first that obtained regulatory approval of an AI-related medical device in Japan, we have experienced unique challenges due to the lack of the established assessment process of AI-medical devices at that time. In 2018, EndoBRAIN (manufactured by Cybernet Systems Co., Ltd., sold by Olympus Corporation), a computer-aided diagnosis (CADx) system compatible with endocytoscopy, a type of magnifying endoscopy, obtained approval as a Class 3 medical device under the Pharmaceutical Affairs Law. Then, multiple CADx and CADe systems, including those from the other companies, were also launched.

EndoBRAIN’s development began in 2013, aiming to create automatic diagnostic software for magnified endoscopic images. The project started as pure scientific research with funding support from the Ministry of Education, Culture, Sports, Science and Technology in Japan. A couple of years later, a, medico-engineering-industrial collaboration was established between Showa University, Nagoya University, and Cybernet Systems Co., Ltd., which enabled an independent project with obtaining regulatory approval as a goal. To obtain approval, consultations with the PMDA were necessary in establishing testing criteria and performance levels. After a year of deliberation, a multicenter study comparing diagnostic accuracy between physicians and EndoBRAIN led to its approval in December 2018.

There have been several regulations and “dramas” behind the scenes. On November 14, 2014, Notification No. 5 of the Pharmaceutical and Food Safety Bureau stated that computer programs which process data obtained from medical devices for diagnosis and treatment are considered medical devices. According to this law, we recognized PMDA approval was required for distributing EndoBRAIN in healthcare venues. We went through several face-to-face consultations with PMDA in understanding legal requirements and facilitating the approval processes. The greatest hurdles that we experienced was the lack of established testing criteria and performance levels to be cleared due to the newly launched law. We needed to consider significant differences in performance criteria depending on the target disease and intended use. For example, whether the AI is intended to detect a disease or to definitively diagnose a disease greatly could affect the evaluation criteria. Therefore, the former may emphasize sensitivity, while the latter may emphasize specificity. We have clarified each of the evaluation points in a tailored way according to the individual consultation with PMDA.

Different from those old days when nothing had been virtually established, important information on the need for clinical testing and performance evaluation criteria has been disclosed by PMDA today (https://www.pmda.go.jp/review-services/drug-reviews/about-reviews/devices/0047.html). First, with regard to the need for clinical testing, the Notification No. 1 of the Pharmaceutical and Medical Devices Evaluation Division (Handling of Performance Evaluation Tests for Diagnostic Medical Devices Using Existing Medical Image Data without Additional Invasive Interventions) specifically provides that if existing data or samples can be collected without additional invasiveness, and if the performance of AI can be evaluated as a result, it does not fall under the category of clinical trials. Additionally, information on the methods and indicators for performance evaluation is also available as part of PMDA’s examination points for SaMD, including product items other than endoscopy, so developers are encouraged to refer to it. We strongly recommend developers to refer to this guidance before launching the project because this can effectively reduce the burden of clearing regulatory approval.

## 4. Reimbursement

Reimbursement is an important step in the widespread use of medical devices in general. Unless there is payment by health insurance bodies for the use of medical devices, it would be very challenging for healthcare venues in buying and using medical devices for patient care. To the best of our knowledge, there had been no reimbursement for the use of AI in colonoscopy in 2023 or before, leading to the very slow implementation of this technology in colonoscopy practice in the world. However, the situation changed in 2024. The Japanese public health insurance body announced the introduction of the added payment as reimbursement for the use of a CADe tool in Japan (EndoBRAIN-EYE ^(9)^) in February of 2024 (https://www.cybernet.co.jp/documents/pdf/news/press/2024/240216E.pdf). We have not seen how this decision affects actual dissemination of the medical device in hospitals and clinic, but it is foreseeable that reimbursement will be a great accelerator for the implementation of a new medical device like an AI tool for colonoscopy.

However, to obtain reimbursement is always a challenge due to the rigorous requirements, which are in fact more challenging than those for regulatory approval acquisition. First, obtaining a regulatory approval is the prerequisite for applying for reimbursement. Second, strong evidence on both clinical effectiveness of the medical device and cost-effectiveness of the device should be presented in a rigorous way in convincing the payers. Third, guideline recommendations by academic societies such as gastroenterology societies could be a great driver of decision-making process in health insurance bodies. Finally, political decision sometimes plays an influential role with regard to introduction of a new reimbursement category, which is completely out of control for developers. This means developers and companies need to pay considerable effort, time, and resources in achieving this goal.

## 5. Possible Downsides after Implementation of AI in Colonoscopy

It was great that this EndoBRAIN-EYE case showed a way in obtaining a reimbursement rate in Japan, which will be referred to when the other manufacturers try to do the same in Japan and in the rest of the world. It is a great motivation for medical industries; however, we also need to understand obtaining regulatory approval and reimbursement of a medical device does not always mean that the approved medical device is beneficial for patient care. There is always a subtle line between benefits and harms of medical intervention, which was not assessed in depth in the history of medical devices in general. Apart from the above-mentioned pragmatic processes of implementation of medical innovation, we as medical researchers and practitioners need to seriously consider what new technology brings to health and society from an objective and long-term perspective. For instance, there have been several unsolved or unanswered questions in CADe in colonoscopy. 1) Does the use of CADe truly reduce colorectal cancers?; 2) Does the use of CADe increase burden of healthcare by increasing polyp removal ^(3), (10)^, frequency of surveillance colonoscopies ^(10)^, and eventually healthcare cost?; 3) Do patients really like to undergo AI-assisted procedures? ^(11)^. 4) Can we expect the same effectiveness for patients with variety of races?

These important questions need to be assessed in a rigorous way, hopefully with literature search using the GRADE scheme, followed by GRADE-based recommendations taking into account patient involvement in the decision-making process ^(12)^. Unfortunately, there have been no such clinical guidelines on AI in colonoscopy for now, allowing the use of AI in colonoscopy without solid clinical guidance now. It is an urgent matter to establish trustworthy guidelines in avoiding unwanted way of using this medical innovation and optimize effectiveness use of the devices. We think medical innovations should be encouraged in general in creating something beneficial for society and people, but we need to strictly assess their effectiveness either during the development process or even after the launch of the products on the market, which may sometimes lead to deimplementation of medical devices. Deimplementation should be a sad story for developers; however, optimization of healthcare quality is actually a history of build and scrap of newly emerged innovation.

## 6. Conclusion

Medical innovation is always exciting in its early stage specifically in creating ideas and piloting preliminary products; however, the road to full implementation of medical devices in clinical practice is accompanied by a lot of processes, bureaucracy, time, and financial requirements. However, it does not mean we should give up developing our brilliant ideas in medicine. The standards are high, or the regulations are strict, but it is important to consult with the regulatory authorities and explore solutions first. In Japan, for example, both PMDA and the Ministry of Health, Labor and Welfare have systems in place where they can be consulted about how to proceed in regulatory approval and reimbursement applications. Several public bodies such as Japan Agency for Medical Research and Development (AMED) open many calls, which financially support regulatory approval processes of medical devices especially when it comes to ambitious challenging projects.

Finally, we need to emphasize the fact that pursuing clinical effectiveness of medical devices or AI devices will not end when we obtain regulatory approval/reimbursement. We encourage continuous effort in uncovering benefits and harms of using such devices, which sometimes takes more time and effort than getting such approvals but eventually contributing to the optimization of healthcare.

## Article Information

### Conflicts of Interest

MM: Olympus Corp. (lecture fees) and Cybernet System Corp. (loyalty fee)

SK: Olympus Corp. (lecture fees) and Cybernet System Corp. (loyalty fee)

YM: Olympus Corp. (lecture fees, consultancy and equipment on loan) and Cybernet System Corp. (loyalty fee)

### Sources of Funding

This work was supported by European Commission (No. 101057099) and Japan Society of Promotion of Science (No. 22H03357).

### Acknowledgement

The authors used ChatGPT, a large language model, for writing assistance of the paper, but it was not used for data collection and interpretation. The authors have carefully reviewed what ChatGPT contributed to and have responsibility for the accuracy of the data presentation and description of the present paper.

### Author Contributions

MM, SK, and YM contributed to conception and design of the work, interpretation of the data for the work, drafting and critical reviewing of the paper, and final approval of the submitted version of the paper. They are responsible for integrity of any part of the work.

### Artificial Intelligence-Assisted Technology

The authors used ChatGPT, a large language model, for writing assistance of the paper, but it was not used for data collection and interpretation. The authors have carefully reviewed what ChatGPT contributed to and have responsibility for the accuracy of the data presentation and description of the present paper.
